# Temperature Dependence of Conduction and Magnetoresistance Properties in Co-TiO_2_ Non-Uniform Nanocomposite Films

**DOI:** 10.3390/nano15221735

**Published:** 2025-11-17

**Authors:** Zhifeng Zhang, Yiwen Zhang, Haoyu Chen, Zhong Wu, Zhenbo Qin, Huiming Ji, Xinjun Liu, Wenbin Hu

**Affiliations:** 1 Key Laboratory of Advanced Ceramics and Machining Technology of Ministry of Education, School of Materials Science and Engineering, Tianjin University, Tianjin 300350, China; 2 State Key Laboratory of Advanced Materials for Intelligent Sensing, Tianjin University, Tianjin 300350, China; 3 Tianjin Key Laboratory of Composite and Functional Materials, Tianjin University, Tianjin 300350, China; 4 Tianjin Key Laboratory of Low Dimensional Materials Physics and Preparation Technology, School of Science, Tianjin University, Tianjin 300350, China

**Keywords:** nanoparticle composite film, magnetoresistance, electron transport mechanism

## Abstract

Co-TiO_2_ materials have rich magnetic and electronic properties for advanced magnetoresistance (MR) sensing field. The non-uniform Co-TiO_2_ nanocomposite films are prepared via magnetron sputtering. With substrate temperature increasing, the particles undergo agglomeration, and this non-uniform structure transits from the superparamagnetic-particle Co distribution to the particle-cluster Co distribution. Consequently, the MR decreases from 6% to 1%, owing to low resistivity. To investigate the electronic transport mechanism, the microstructural analysis and temperature-dependent fitting calculations of conduction and MR were investigated. In this study, non-uniform nanocomposite films with a broad particle size distribution were fabricated. With testing temperature decreasing, electron transport changes from higher order hopping to higher order cotunneling processes. The non-uniform films deposited at room temperature exhibited a negative MR up to 30% at 2 K, which was attributed to higher order cotunneling in the Coulomb blockade regime and explained by establishing a non-uniform multi-channel conduction model.

## 1. Introduction

Magnetic nanocomposite thin films [1,2,3,4,5,6,7,8,9] consisting of ferromagnetic metal nanoparticles dispersed in a ceramic oxide matrix have attracted significant attention due to their unique combination of magnetic and electronic properties. In this systems, metallic nanoparticles such as Co, Fe, or Ni are uniformly embedded within an insulating or semiconducting oxide matrix (e.g., Al_2_O_3_, TiO_2_, or SiO_2_), forming a granular structure at the nanometer scale. The strong spatial confinement of conduction electrons and the presence of numerous metal–oxide interfaces give rise to various spin-dependent transport phenomena, including tunneling magnetoresistance (TMR), spin-dependent hopping, and cotunneling effects. The ceramic-based granular films exhibit high thermal stability, well-defined microstructures, and controllable magnetic coupling between particles, making them ideal candidates for studying spin-polarized transport and developing spintronic devices [10,11,12,13,14,15,16,17,18,19,20,21]. Furthermore, the electrical conduction in such films is highly sensitive to temperature, magnetic field, and microstructural parameters (such as particle size and interparticle separation), leading to rich physical electron transport behaviors that bridge the gap between metallic and insulating regimes.

TiO_2_ is an n-type wide-bandgap (3.2 eV) semiconductor material, whose electrons can be transported in the form of tunneling. A TMR effect has been found in Co-TiO_2_ nanocomposite granular films [5]. Previous studies have made significant contributions to the investigation of the Co-Ti-O composite system. The preparation of Co-Ti-O system nanocomposite granular films has been achieved by methods including magnetron co-sputtering [22], pulsed laser deposition [23], electrochemical deposition [24], and reactive sputtering [25]. Due to the low crystallinity of magnetic metals and the accompanying partial oxidation, it is difficult for Co-TiO_2_ nanocomposite films to achieve high saturation magnetization and TMR property.

In a previous study [5], by increasing the substrate temperature, the saturation magnetization increased from 0.13 to 0.43 T. However, conductive paths form between particle clusters, which induce a decrease in resistivity from 1600 to 76 μ Ω m. This causes the room-temperature (RT) TMR to decrease almost linearly from 6% to 1%. Therefore, the electronic transport mechanism is important to study for understanding this change in TMR. In TiO_2_–metal composite systems, various conduction mechanisms have been investigated, such as tunneling, hopping, Schottky or Poole–Frenkel mechanisms [26], Mott’s law [27], or other mechanisms. However, for Co-TiO_2_ non-uniform nanocomposite films, the mechanism underlying electronic transmission has not been sufficiently explored. In this paper, through the investigation of influencing factors in the electronic transport processes of Co-TiO_2_ non-uniform nanocomposite films at low temperature region to room temperature (2–300 K), the relationships between magnetic-electrical properties, and microstructure in Co-TiO_2_ nanocomposite films are further revealed the conduction mechanism based on the experimental results and the previous studies, a possible non-uniform multi-channel conduction model is proposed to further understand the electron transport mechanism in Co-TiO_2_ nano-composite films. which include conduction mechanisms such as high order cotunneling, high order hopping, superparamagnetic, and metallic conduction pathways, similar to those widely reported in other nanoparticle-based films [10,12,14,17].

## 2. Materials and Methods

The Co-TiO_2_ nanoparticle composite films were fabricated using a four-target high-vacuum magnetron sputtering system (Shenyang Scientific Instrument Co., Ltd., Chinese Academy of Sciences, Shenyang, China). A metallic Co target (>99.95 at.%) was sputtered with a strong-magnetic DC source, while a TiO_2_ target (>99.95 at.%) was sputtered with a permanent-magnetic RF source. The Co target was positioned at the bottom of the chamber (19 cm from the substrate), sputtering upward, and the TiO_2_ target was placed at the side (14 cm from the substrate), sputtering diagonally. During the deposition process, the argon gas flow rate was set to 20 sccm, and the working pressure during sputtering was maintained at 2.2 Pa. The substrate rotation speed was 4 revolutions per minute (rpm). The sputtering powers for the Co and TiO_2_ targets were set to 50 W and 150 W, respectively. Prior to the main deposition, both targets were pre-sputtered for several minutes to remove surface oxides formed on the target materials. During the sputtering process, the substrate temperatures were set to RT, 150 °C, 300 °C, and 400 °C, respectively. The deposition time for each film was controlled at 1 h, resulting in an average film thickness of approximately 160 nm [5].

For the characterization of the experimental materials, the microstructural composition was mainly examined using a transmission electron microscope (TEM, JEM-2100F, JEOL, Tokyo, Japan), while the surface morphology was analyzed with a field emission scanning electron microscope (FESEM, JSM-7800F, JEOL, Tokyo, Japan). The electrical and MR properties of the samples were measured at low temperature region to room temperature (2–300 K) using a physical property measurement system (PPMS-9, Quantum Design, San Diego, CA, USA).

## 3. Composition and Morphology

**Figure 1:**
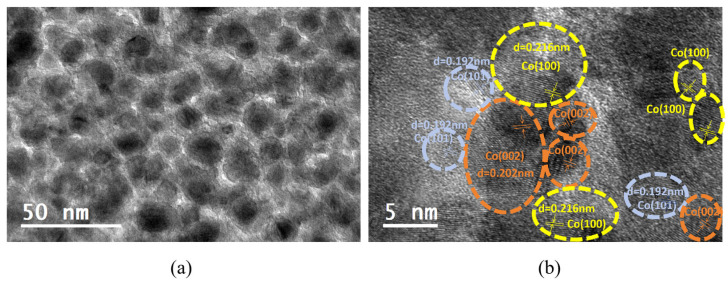
The TEM image (**a**) and HRTEM image (**b**) for Co-TiO_2_ non-uniform nanocomposite films deposited at RT.

Figure 1 shows the transmission electron microscopy (TEM) image of Co-TiO_2_ non-uniform nanocomposite films prepared at room-temperature substrate conditions [28]. As observed in Figure 1a, the nanoparticles in the film have sizes ranging from 3 to 10 nm. Most of the particles are isolated from each other, while a small portion are interconnected. In the high-resolution TEM (HRTEM) image of Figure 1b, clear lattice fringes can be identified, with interplanar spacings of 0.216 nm, 0.202 nm, and 0.192 nm (The arrows in the figure indicate the interplanar spacing), which correspond to the (100), (002), and (101) planes of hexagonal Co, respectively. This result is consistent with X-ray diffraction peaks appeared at 41.5°, 44.4°, and 47.4° in the previous report [5]. Moreover, the regions surrounding the Co nanoparticles without visible lattice fringes indicate the amorphous TiO_2_ matrix. This demonstrates that most crystalline Co particles in the film are encapsulated by the amorphous TiO_2_ matrix [29]. This non-uniform nanocomposite films show a broad particle size distribution.

## 4. Results

**Figure 2:**
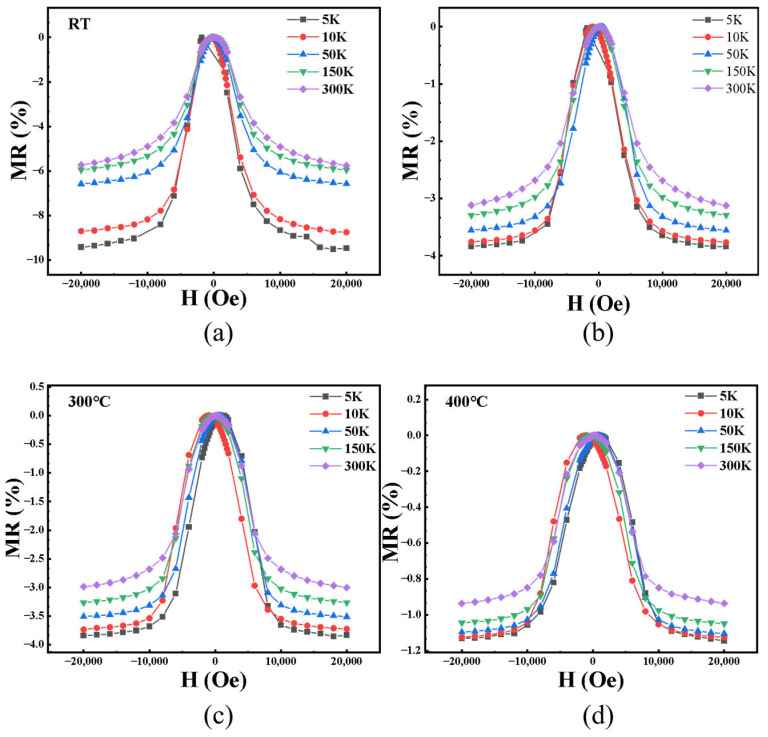
The MR behavior of the samples prepared at different substrate temperatures under various measurement temperatures: (**a**) RT; (**b**) 150 °C; (**c**) 300 °C; and (**d**) 400 °C.

**Figure 3:**
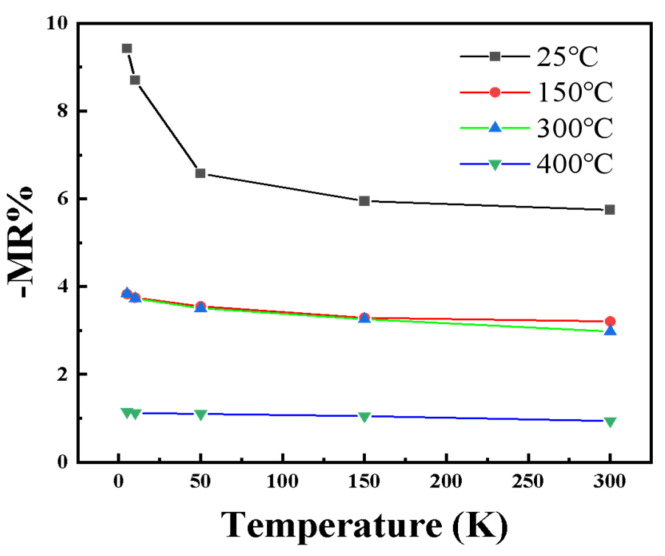
Trend lines of MR versus measurement temperature at different substrate temperatures.

Figure 2 illustrates the magnetoresistance (MR) behavior of the samples prepared at different substrate temperatures under various measurement temperatures. As the temperature increases, magnetic metal particles in the films tend to cluster, and partial contact between particles occurs. This leads to the local formation of conductive pathways, resulting in a gradual transition of the electron transport mechanism from tunneling conduction to metallic conduction [30,31]. Meanwhile, the MR ratio decreases almost linearly from about 6% to 1% with increasing substrate temperature, indicating a strong correlation between MR behavior and the underlying electron transport mechanism. In Figure 3, it is observed that the MR of all samples decreases with decreasing measurement temperature. A common trend is observed: among the four samples, the negative magnetoresistance with RT substrate temperature is the highest, and its increasing trend of −MR value is the most pronounced.

**Figure 4:**
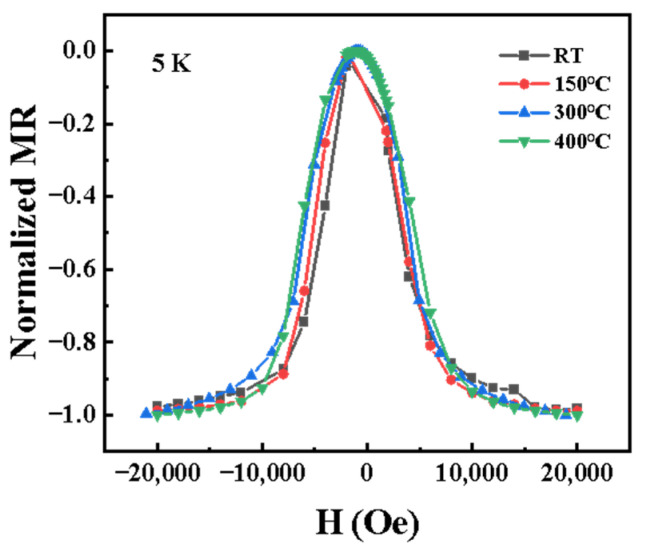
Normalized magnetoresistance (MR) curves at 5 K for samples deposited at substrate temperatures of RT, 150 °C, 300 °C, and 400 °C.

In Figure 4, the normalized MR curves for samples prepared at different substrate temperatures tested at 5 K are shown. The peak width of the normalized MR curves gradually broadens with increasing substrate temperature, owing to the formation of non-uniform structures with size-distributed ferromagnetic Co clusters [17]. These observations are consistent with dependence of MR’s external magnetic field at various substrate temperature tested at room temperature, which is explained by the XRD and *M*-*H* fitting analysis reported previously [5].

The essence of MR lies in the changes of electron transport under an applied magnetic field, which in turn alters the resistivity of the material system. Therefore, a thorough investigation of the electron transport mechanisms in nanocomposite thin films, and their correlation with microstructural evolution, is crucial for a deeper understanding of the MR formation process and for enhancing the MR performance of the system.

**Figure 5:**
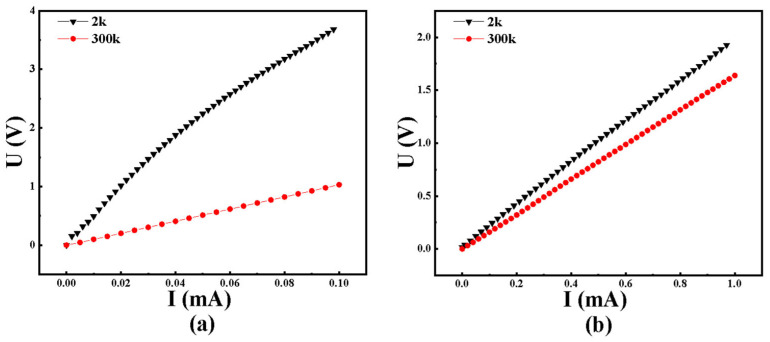
U-I curves at different temperatures when sputtering temperatures were at (**a**) room temperature and (**b**) 400 °C.

As shown in Figure 5a, the current-voltage (*I*–*V*) curve of the film prepared at room temperature exhibits a linear dependence (red line, measured at 300 K), indicating that the film follows an ohmic conduction mechanism at room temperature. However, when the temperature is reduced to 2 K (black line), the *I*–*V* curve becomes nonlinear, suggesting a transition from ohmic conduction at high temperature to non-ohmic conduction at low temperature.

Figure 5b presents the *I*–*V* characteristics of the film prepared at a substrate temperature of 400 °C. In this case, the voltage-current dependence remains linear at both 300 K and 2 K, indicating that the conduction mechanism of the high-temperature deposited film remains ohmic and does not change with measurement temperature.

From Figure 5a,b, it can also be observed that the resistance values of the films, regardless of whether they were prepared at room temperature or at 400 °C, increase significantly as the measurement temperature decreases. This behavior reflects a typical non-metallic characteristic, where electron transport is dominated by tunneling conduction and hopping conduction mechanisms.

**Figure 6:**
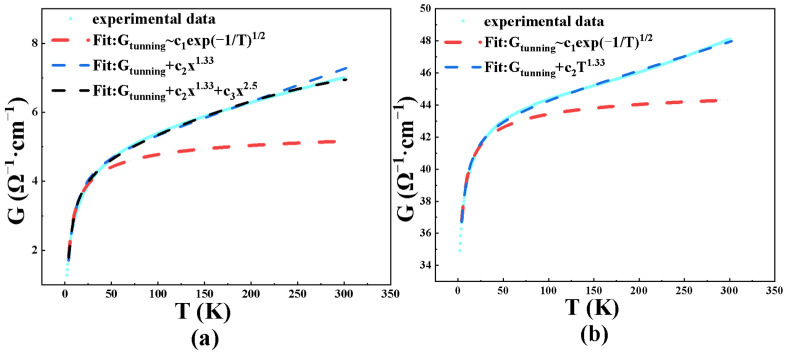
The variation of conductance with temperature in preparing films at (**a**) room temperature and (**b**) 400 °C.

To understand the electron transport mechanisms in Co-TiO_2_ nanoparticle composite films, the temperature-dependent conductance was measured for the films prepared at different substrate temperatures, as shown in Figure 6. The nanoparticle composite films exhibit two types of metallic conduction mechanisms [32]: at high temperatures, electron transport is dominated by spin-independent conduction, called high order hopping mechanism; whereas at low temperatures, spin-dependent conduction between particles prevails, called high order cotunneling mechanism. This theory was first proposed by Glazman and Matveev [33]. By combining these two conduction mechanisms, the overall conductance can be expressed as the folloiwng:

(1) $$G=G_{1}+G_{2}=c_{1}\exp[\left(-\frac{T_{0}}{T}\right)]+\sum c_{N}T^{\gamma }$$

(2) $$\gamma =N-(\frac{2}{N+1})$$

where $c_{1}$ and $c_{N}$ are fitting parameters, and their values vary with the order of higher order hopping, and *N* represents the number of localized states, $c_{N}$ is a free fitting parameter, and *G* is the conductance. Notice the value of *γ* is not continuous. In the case of the second-order hopping (*N* = 2), *G* ∝ *T*^1.33^. For the third-order hopping (*N* = 3), *G* ∝ *T*^2.5^, and for the fourth-order hopping (*N* = 4), *G* ∝ *T*^3.6^.

Figure 6a shows the temperature-dependent conductance of the film prepared at room temperature. Below 35 K, the spin-dependent conduction mechanism fits the experimental data well. As the temperature increases, the data gradually deviate from the spin-dependent tunneling mechanism. In the range of 35–250 K, the experimental data can be accurately fitted by introducing a second-order spin-independent hopping conduction mechanism. When the measurement temperature exceeds 250 K, the electron transport gradually transitions from a second-order hopping mechanism to a third-order spin-independent hopping conduction mechanism.

For films prepared at a substrate temperature of 400 °C, as shown in Figure 6b, a similar trend is observed. In the range of 2–30 K, the conductance can be well described by the spin-dependent mechanism, while in the range of 30–300 K, introducing the second-order spin-independent hopping mechanism enables an accurate fit to the data. The increase in temperature facilitates higher order chain-like hopping of electrons along localized states in the nanoparticle composite films [34].

By comparing Figure 6a,b, it is evident that with increasing substrate sputtering temperature, the upper temperature limit for spin-dependent tunneling conduction decreases significantly. Furthermore, at higher temperatures, the spin-dependent tunneling mechanism gradually transitions to a higher order spin-independent hopping conduction mechanism, which will be discussed in non-uniform multi-channel conduction model.

**Figure 7:**
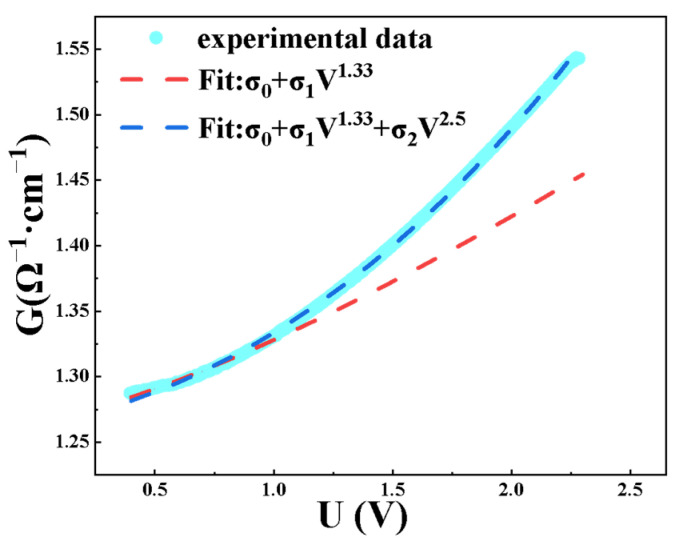
Variation rule of conductance with bias voltage and corresponding data fitting.

To further investigated the non-ohmic conduction mechanism exhibited by the films prepared at low temperatures, the data fitting analysis was performed on the film conductance as a function of bias voltage at 2 K. Figure 7 shows the conductance–bias voltage curve. Increasing the bias voltage allows more localized spin states to participate in conduction. The higher order hopping theory proposed the inelastic hopping through two or more localized states (*N* ≥ 2) involving electron-phonon interaction, in which a power-law dependence of temperature has been given and the relationship between conductance and bias voltage also follows a power-law behavior and can be fitted by the following equation [25,32]:

(3) $$G=\sigma_{0}+\sigma_{1}V^{1.33}+\sigma_{2}V^{2.5}$$

where $\sigma_{0}$, $\sigma_{1}$, and $\sigma_{2}$ are free parameters. Better fits at the high-bias end can be obtained if one includes a higher order term of *V*^3.6^.

When the bias voltage is below 1 V, the electron transport is well described by a second-order hopping mechanism. As the bias voltage increases to the range of 1–2.5 V, the conduction mechanism gradually transitions from second-order to third-order hopping. This indicates that increasing the bias voltage facilitates electrons to perform chain-like hopping through a greater number of localized states [35], resulting in the nonlinear dependence of conductance on bias voltage.

**Figure 8:**
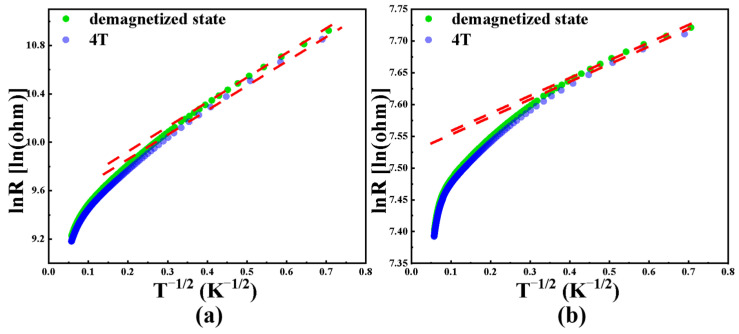
The change rule of ln*R* with *T*^−1/2^ and the corresponding data fitting (substrate temperature was room temperature *R*_0_ = 12,000 Ω, $8e^{2}\alpha /\varepsilon k_{B}$ = 0.066 K, $8\alpha J/k_{B}$ = 5.01 K (**a**) and 400 °C *R*_0_ = 1900 Ω, $8e^{2}\alpha /\varepsilon k_{B}$ = 5.21 × 10^−4^ K, $8\alpha J/k_{B}$ = 0.0544 K (**b**)).

To better understand the non-metallic conduction of the films at low metal content, the resistance–temperature relationship was transformed into a plot of ln*R* versus *T*^−1/2^. Figure 8a corresponds to the resistance–temperature behavior of the film prepared at room temperature, while Figure 8b corresponds to the film prepared at a substrate temperature of 400 °C. The analysis reveals that a good linear relationship between ln*R* and *T*^−1/2^ can be observed below 12 K and 6 K for the two cases, respectively, and this relationship can be fitted using equation [36,37,38,39]:

(4) $$\ln R_{ij}=\ln\left[R_{0}/(1+P^{2}\cos\theta )\right]+{T_{0}}^{1/2}T^{-1/2}$$

where *R*_ij_ denotes particle resistance between i and j particles, *R*_0_ denotes initial resistance, *P* denotes spin polarization, *θ* denotes angle between magnetization directions, *T*_0_ denotes initial temperature, and *T* denotes temperature.

When there is no external magnetic field, the cosine of magnetization angle cos*θ* can be regarded as 0, and the formula can be simplified accordingly:

(5) $$\ln R=\ln R_{0}+(\frac{8e^{2}\alpha }{\varepsilon k_{B}}{)}^{1/2}T^{-1/2}$$

where *R* denotes material resistance, 1/*α* denotes spin localization length, and *k*_B_ denotes Boltzmann constant.

When a sufficiently strong external magnetic field is applied, the magnetization directions of the magnetic particles in the film tend to align, so that the cosine of the magnetization angle, cos*θ*, can be considered as one. In this case, the formula can be rewritten as follow:

(6) $$lnR=lnR_{0}{-\ln(1+p}^{2})+(\frac{8e^{2}\alpha }{\varepsilon k_{B}}-\frac{8\alpha J}{k_{B}}{)}^{1/2}T^{-1/2}.$$

Regardless of whether an external magnetic field is applied or not, the parameters ln*R* and T^−1/2^ exhibit a linear relationship. As the temperature increases, the experimental values gradually deviate from the fitted curves, due to higher order electron hopping [36]. $8e^{2}\alpha /\varepsilon k_{B}$ and $8\alpha J/k_{B}$ are fitting constants summarized under Figure 8. In Co-TiO_2_ nanoparticle composite films, with increasing substrate temperature, the initial *R*_0_ resistance decreases from 12,000 Ω to 1900 Ω. This corresponds to the agglomeration and growth of metallic particles in the films. The resistance change pronounced seriously, indicating that temperature adjustment primarily affects the microscopic state of magnetic metal. Furthermore, the upper temperature limit for tunneling-related conduction decreases from 12 K to 6 K with increasing substrate temperature. Changing the substrate temperature can significantly modify the electrical properties of the nanoparticle composite films and the electron tunneling mechanism at low temperatures.

Changing the substrate temperature can significantly modify the electrical properties of the nanoparticle composite films and the electron tunneling mechanism at low temperatures.

With decreasing temperature, the Coulomb blockade effect between magnetic nanoparticles becomes significant, promoting higher order cotunneling processes between particles of different sizes. Meanwhile, the spin-independent hopping conduction mechanism is gradually suppressed. These effects collectively lead to the exponential increase of MR with decreasing temperature. The experimental MR ratios are lower than the calculated MR values, indicating that the cotunneling process alone does not result in such MR ratio deviation.

In the nanoparticle composite films, the metallic particles possess Coulomb charging energy, which is proportional to the particle size. During the transport process, electrons preferentially tunnel between larger particles with lower charging energy. However, as the temperature decreases, thermally activated hopping transport is suppressed, and the conduction mechanism shifts toward spin-dependent tunneling. Under the condition of minimum energy loss, electrons tend to tunnel from smaller metallic particles to larger ones, thereby forming higher order cotunneling processes [39].

**Figure 9:**
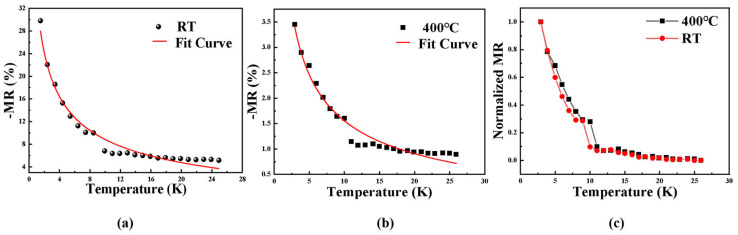
The variation curve of magnetoresistance at low temperature and corresponding data fitting (**a**) m^2^p^2^ = 0.05884, *E_c_*/8*ks* = 68.46178 K; (**b**) m^2^p^2^ = 0.00668, *E_c_*/8*ks* = 112.38706 K; and (**c**) normalized magnetoresistance (MR) curves for samples deposited at substrate RT and temperature.

At room temperature, the MR of the material is mainly governed by the superparamagnetic mechanism, whereas at low temperatures, the high order cotunneling model provides an explanation for the increase in MR observed in the Co–TiO_2_ non-uniform nanocomposite films. To quantitatively describe the cotunneling conduction mechanism of the films at low temperatures, In Figure 9, the transport behavior of the films was fitted. The higher order cotunneling model can be expressed as the following [25,40]:

(7) $$MR=\Delta \rho /\rho =1-{\left(1+m^{2}P^{2}\right)}^{-(n^{*}+1)}$$

(8) $$n^{*}={\left(E_{c}/8ksT\right)}^{1/2}$$

where *E_c_* denotes the Coulomb charging energy, *k* is a constant, *s* represents the average distance between particles, m is the normalized magnetization, and *P* is the spin polarization. In the temperature range of 2 K to 25 K, the variation of MR with temperature fits well with the higher order cotunneling model. This indicates that the films possess a broad particle size distribution, where some larger metallic particles may transport electrons via smaller particles, thereby forming a higher order cotunneling conduction mechanism, which in turn leads to the sharp increase in a −MR at low temperatures. Below 10 K, the experimental magnetoresistance of the material fits well with the high order cotunneling model. At this stage, the high order cotunneling mechanism becomes dominant owing to electronic transmission through multiple smaller Co particles. In Figure 9c, the normalized magnetoresistance (MR) curves at low temperatures for the RT and 400 °C samples are shown. As the testing temperature decreases, both the curves show similar trends of change. Around 10 K, the experimentally measured −MR value suddenly drops and deviates from the fitting curve, which should be attributed to the fact that the PPMS system requires approximately 30 min of thermal stabilization at 10 K. Insufficient stabilization may lead to deviations in the measured magnetoresistance. As the measurement temperature increases, the experimental curves deviate significantly from the high order cotunneling fitting curve. Moreover, the magnetic moments can fluctuate freely, the superparamagnetic conduction pathway becomes dominant for MR, and thermally activated spin-independent hopping contributes to most of the conductivity.

**Figure 10:**
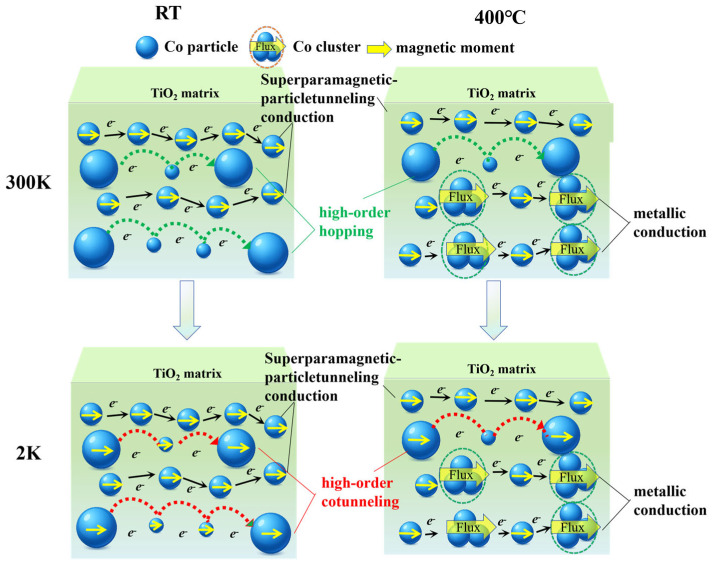
Schematic diagram of non-uniform multi-channel conduction model of Co-TiO_2_ non-uniform nanocomposite films at RT and 400 °C substrate temperature.

To explain these phenomena, the non-uniform multi-channel conduction model has been proposed in Figure 10. the blue ball represents Co particles, while the surrounding green regions indicate the TiO_2_ matrix.

At low substrate temperatures, Co nanoparticles maintain relatively small sizes and are well isolated by the amorphous TiO_2_ matrix, exhibiting a superparamagnetic-particle Co distribution. The arrows in the figure represent the conduction processes of different transport mechanisms: the black arrows indicate the superparamagnetic conduction process, the red arrows indicate the higher order cotunneling process, and the green arrows indicate the higher order hopping process. At a testing temperature of 300 K, there exist tunneling conduction pathways through the superparamagnetic particles, which is defined as the superparamagnetic tunneling conduction mechanism. Due to the non-uniform structure of the film, there are large metallic particles that are far apart; electron transport between them can occur via high order hopping through intermediate small Co particles, which is defined as the high order hopping conduction mechanism. As the testing temperature decreases to 2 K, the conduction mechanism changes from thermally activated spin-independent hopping to spin-dependent cotunneling, which causes the −MR value to increase sharply to 30% at 2 K.

At high substrate temperature, the particle size increases, and agglomeration occurs, which leads to the formation of Co clusters. The emergence of these clusters introduces a metallic conduction mechanism in the sample, which significantly reduces the room temperature MR value. At a testing temperature of 300 K, the sample is dominated by metallic conduction, with less contributions from superparamagnetic particle tunneling conduction pathways and high order hopping conduction mechanisms. As the testing temperature decreases to 2 K, metallic conduction pathways remain dominant, with a less amount of spin-dependent cotunneling conduction.

## 5. Conclusions

The non-uniform nanocomposite films with a broad particle size distribution consist hexagonal Co nanoparticles embedded in amorphous TiO_2_. Films with high substrate temperature showed lower MR owing to metallic conduction pathways formed, which induce the metallic conduction mechanism at 2 and 300 K measurement temperature. For films deposited at room-temperature, higher order cotunneling in the Coulomb blockade regime causes a sharp MR increase at low temperatures, reaching 30% at 2 K. As measurement temperature rises from 2 to 300 K, conduction changes from spin-dependent tunneling to thermally activated spin-independent hopping. A speculated non-uniform multi-channel conduction model has been established, which provides a direction for the development of high-performance spintronic devices based on heterogeneous nanocomposite films.

## Data Availability

Data are contained within the article.
